# Anchoring Pd-nanoparticles on dithiocarbamate- functionalized SBA-15 for hydrogen generation from formic acid

**DOI:** 10.1038/s41598-020-75369-y

**Published:** 2020-10-23

**Authors:** Mustafa Farajzadeh, Hassan Alamgholiloo, Fariba Nasibipour, Reza Banaei, Sadegh Rostamnia

**Affiliations:** 1 Organic and Nano Group (ONG), Department of Chemistry, Faculty of Science, University of Maragheh, PO BOX, 55181-83111 Maragheh, Iran; 2 Organic and Nano Group (ONG), Department of Chemistry, Iran University of Science and Technology (IUST), PO Box, 16846-13114 Tehran, Iran

**Keywords:** Energy science and technology, Renewable energy, Catalysis, Green chemistry, Materials chemistry

## Abstract

Hydrogen (H_2_) generation from natural biological metabolic products has remained a huge challenge for the energy arena. However, designing a catalytic system with complementary properties including high surface area, high loading, and easy separation offers a promising route for efficient utilization of nanoreactors for prospective H_2_ suppliers to a fuel cell. Herein, selective dehydrogenation of formic acid (FA) as a natural biological metabolic product to H_2_ and CO_2_ gas mixtures has been studied by supporting ultrafine palladium nanoparticles on organosulfur-functionalized SBA-15 nanoreactor under ultrasonic irradiation. The effects of the porous structure as a nanoreactor, and organosulfur groups, which presented around the Pd due to their prominent roles in anchoring and stabilizing of Pd NPs, studied as a superior catalyst for selective dehydrogenation of FA. Whole catalytic systems were utilized in ultrasonic irradiation in the absence of additives to provide excellent TOF/TON values. It was found that propose catalyst is a greener, recyclable, and more suitable option for the large-scale application and provide some new insights into stabilization of ultra-fine metal nanoparticle for a variety of applications.

## Introduction

It is clear that fossil fuel reserves are limited and some environmental problems such as ozone depletion, acid rain, and some other issues in this case that is arising from the presence of CO_2_, NO_x,_ and SOx in the publication gases, ensue because of consuming coal, natural gas and petroleum^1,2^. Most of these problems can be crossed by using clean and renewable energy sources. Hydrogen (H_2_) is the globally accepted and most suitable and clean energy source, which could solve the world energy problem and diminish the environmental pollution emanated from fossil fuels^3^. Formic acid (HCOOH, FA) is an appropriate liquid for storage and hydrogen production because it normally is non-toxic, available from some process like the reduction of carbon dioxide or biomass processing, and has a significant content of hydrogen (4.4 wt%) at room temperature^4–6^. Besides, FA can readily release H_2_ under room temperature in the presence of homogeneous and heterogeneous catalysts under the reaction: HCOOH → CO_2_ + H_2_.

Recently, tremendous efforts have been made for selective dehydrogenation of FA with homogeneous^7–14^ and heterogeneous catalysts^15–24^, which have their own unbeatable advantages. Despite the homogeneous catalysts based on Ir, Ru, and Fe organometallic complex usually have great catalytic activity and selectivity, while gold^25^, Platinum^26^, and palladium^4^ well known heterogeneous catalysts used in the decomposition of FA. However, some reports have shown that nanostructure with base Pd such as Au_0.5_Pd_0.5_/NH_2_-N-rGO^27^, Pd-MIL-125^28^, Pd_1_Au_1_/30-LA^29^, AP-SiO_2_@PDA-NGO@Pd^30^, PdAu-MnOx/N-SiO_2_^31^, CrAuPd/N-SiO_2_^32^, IrPdAu/NH_2_-SBA-15^33^, Ag_0.2_Au_0.4_Pd_0.4_/rGO^34^, Au@Pd/UiO-66(Zr_x_Ti_y_)^35^, Au–Pd/MIL-101^36^, and PdCoNi/TiO_2_^37^, can efficiently decomposed FA under neat condition. Recently, our research group explored the catalytic application of Pd@Cu-MOF^38^ and Pd@Cr-MOF^39^ with open metal sites as an ideal Pd-catalyst for H_2_ generation from FA in neat condition. Despite the inspiring progress, selective dehydrogenation of FA still suffers from some significant limitations such as low conversion, highly Metal-dependent, and large energy consumption.

Ultrasonic technology is a safe and efficient new treatment method for the chemical transformations and environmental arena. This technology has advantages such as high efficiency, short reaction time due to local temperature, high pressure and cooling speed^40–44^. However, despite the unique ultrasonic technology and their effect in catalytic systems for energy arena such as selective dehydrogenation has been unexplored to date. However, despite the unique ultrasonic technology and their effect in catalytic systems for energy arena such as selective dehydrogenation has been unexplored to date. On the other hand, dithiocarbamate (DTC) ligands possess well coordination capability, versatile coordination modes, and unique chemical properties of their metal complexes and nanoparticles^45–48^. The best affinity between the DTC ligand and the template walls is basically important for grafting and stabilizing the Pd NPs, and inhibition of the "Ostwald Ripening" process. Based on the above considerations, we envisioned an economical method for the bifunctional fabrication catalyst with features such as benign environmental, stable, and affordable for selective dehydrogenation of aqueous solutions of HCOOH We exploited both metal and organosites for the sustainability for H_2_ generation from FA. The resultant of Pd_NPs_@SBA-15/DTC with uniform pores exhibit excellent catalytic activity in the presence of ultrasonic wave as a synergistic agent with the turnover frequency (TOF) value of 1952 h^−1^, turnover number (TON) value of 3904 and 9.86 mL of gas (H_2_ and CO_2_) was obtained in 120 min.

## Experimental

### Materials and methods

Tetraethyl orthosilicate (TEOS, Aldrich, 99.8%), poly(ethylene glycol)-block-poly(propylene glycol)-block-poly(ethylene glycol) (P123, Aldrich), 3-aminopropyl triethoxysilane (Merck, 99.9%), PdCl_2_ (Merck, 99.5%), CS_2_ anhydrous (Aldrich, ≥ 99%), Formic acid (Aldrich, ≥ 95%), ethanol (Merck, 98.5%), CH_3_CN (Merck, 99.5%), and methanol (Merck, 99.5%) were used without further purification. Ultrapure water with the specific resistivity of 18.5 MΩ cm was used for all experiments.

### Characterization

The FT-IR spectra were performed using a PerkinElmer-Spectrum Two with ATR probe. The morphologies of the samples were taken on FE-SEM, Zeiss EM 960 A. The TEM images were recorded on Zeiss-EM10C-100 kV. The volume of gases (H_2_ + CO_2_) analyzed by a gas chromatograph (GC) and negligible carbon monoxide (CO) evolution was observed. The results compared with obtained information from the automatic sensor of H_2_ and CO_2_ gases.

### Preparation of SBA-15/DTC

SBA-15 nanoreactor was synthesized a supramolecular self-assembly strategy according to our previous method^49,50^ and other reports^51–53^. 1.0 g of calcined SBA-15 was dried at 120 °C under vacuum for 6 h. After this, the solid material was dispersed in 25 mL of dried-toluene under inert atmosphere and after adding 2 mmol of 3-aminopropyl triethoxysilane (APTES) to the mixture refluxed for 2 h at 110 °C. Finally, the solid white product was separated and washed multiple times by DI-water and methanol. Then, CS_2_ (1 mmol) was added to the mixture with a few drops NH_3_ and stirred at ambient condition for 12 h. Finally obtained (*SBA-15/DTC*) was collected and washed with EtOH and then dried for 5 h at 70 °C.

### Preparation of Pd_NPs_@SBA-15/DTC

PdCl_2_ (100 mg) was dissolved in DI-H_2_O and CH_3_CN (1:1 v/v, 5 mL) and this solution was added to a suspension of SBA-15/DTC (500 mg) in EtOH (25 mL). After stirring for 2 h at ambient condition, the resulting solid material was collected, washed 2 times with EtOH, 1 time with CH_2_Cl_2_ and dried at room temperature for 12 h. Then the solid result was dispersed in 10 mL MeOH and a solution 10 mM of NaBH_4_ was added dropwise. After stirring for 2 h at ambient condition, the final solid result was collected by filtration, washed with MeOH and CH_2_Cl_2_ for several times and dried at room temperature. According to atomic absorption analysis (AAS) of the Pd-catalyst, the amount of loaded Pd was 0.010 mmol per gr of catalyst.

### Catalytic dehydrogenation of FA

Catalytic dehydrogenation of FA was accomplished in a two-necked round bottom flask (volume, 80 mL). One neck of the flask is connected to a gas burette and the other was reserved for adding 50 mg of Pd-catalyst mixed with 5.0 ml of FA-SF ( FA: 5% v/v with a mole ratio of 1:1) under ultrasonic irradiation. To examine whether or not CO gas was formed in the dehydrogenation process, the generation gases analyzed by GC and NaOH trap experiment. Using 6.64 mmol of FA under ultrasonic irradiation, the complete conversion is expected to develop 9.86 mL of the gases mixture with TOF value of 1952 h^-1^, as calculated from the following equation.

$$TOF_{initial} = \frac{{{{P_{atm} V_{gas} } \mathord{\left/ {\vphantom {{P_{atm} V_{gas} } {RT}}} \right. \kern-\nulldelimiterspace} {RT}}}}{{2n_{pd} t}}$$

TOF was obtained from decomposition of FA/SF (1:1), which *Patm* is atmospheric pressure, *Vgas* is volume of produced gas, *R* is universal gas constant, *T* is room temperature and *n*_Pd_ is mole number of Pd in the catalyst.

## Results and discussion

**Figure 1:**
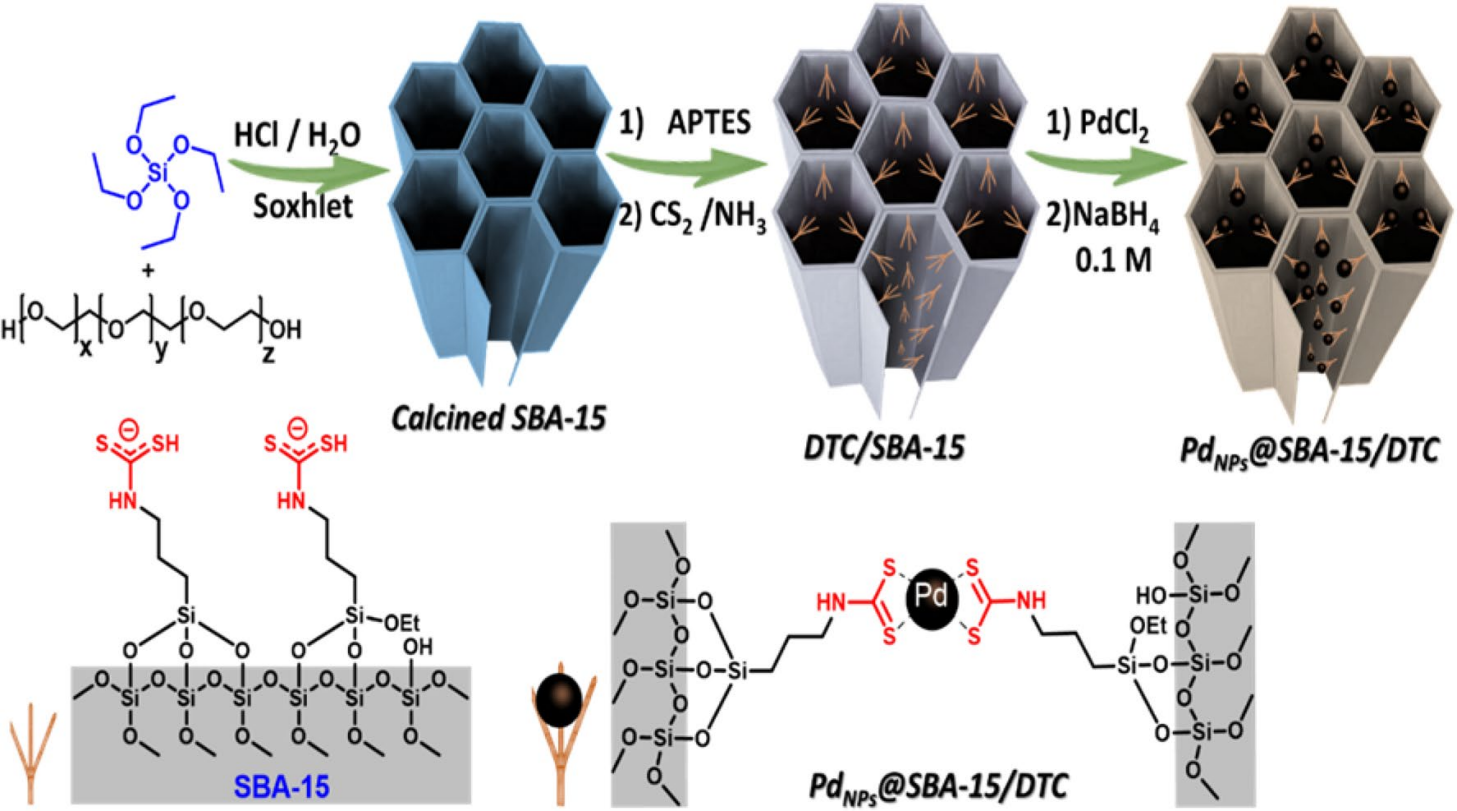
Schematic illustration for preparation of the Pd_NPs_@SBA-15/DTC.

In this study, SBA-15 was prepared a supramolecular self-assembly strategy and then dithiocarbamate grafted on 3D ordered mesoporous silica. The resulted white powder was then utilized as the excellent support for immobilizing ultra-fine Pd NPs Fig. 1. The hydrophobic-hydrophilic properties of the mesoporous silica are controlled By embedding the DTC ligand into the pore walls. Plus, the DTC ligand and pores structure of nanoreactor SBA-15 has a vital role in the excellent control of Pd metal size and homogeneous dispersion, of that, then boosting the catalytic activity for selective dehydrogenation of FA.

**Figure 2:**
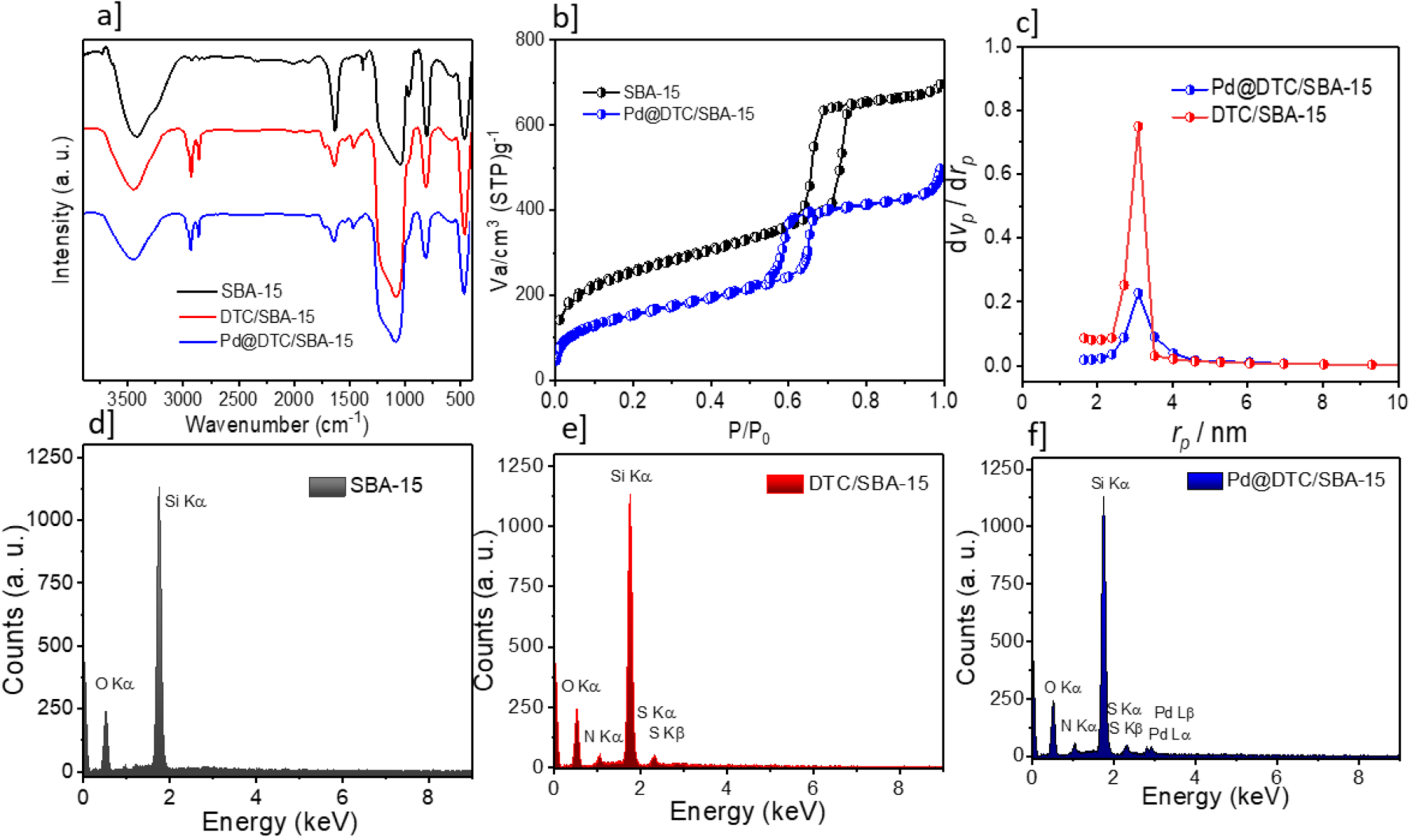
(**a**) FT-IR spectrum and (**d**–**f**) Eds analysis of SBA-15, SBA-15/DTC, Pd_NPs_@SBA-15/DTC. (**c**,**b**) BET and BJH analysis of SBA-15/DTC, Pd_NPs_@SBA-15/DTC.

The chemical groups of the SBA-15, DTC/SBA-15, and Pd_NPs_@SBA-15/DTC were analyzed by FTIR spectra. As depicted in Fig. 2a, several absorption bands in 1400–1700 can be ascribed to ligand DTC in the SBA-15/DTC framework. The signal appears in 3241 cm^−1^ is attributed to N–H stretch mode, which suggesting the successful preparation of SBA-15/DTC. The specific surface areas of SBA-15/DTC and Pd_NPs_@SBA-15/DTC were measured via the BET method. As shown in Fig. 2b, all the catalysts exhibited type IV isotherms, reflecting the presence of the meso-size pores. The corresponding pore diameter distributions for SBA-15/DTC and Pd_NPs_@SBA-15/DTC were 3.39 nm and 3.17 nm, respectively (Fig. 2c). Also, all DTC ligand (Fig. 2e) and Pd particles (Fig. 2f) are well-anchored onto mesoporous silica (Fig. 2d). The existence of all constituent elements (i.e., Si, C, O, N, S, and Pd) in structures of Pd_NPs_@SBA-15/DTC has been proved by the EDS analysis.

**Figure 3:**
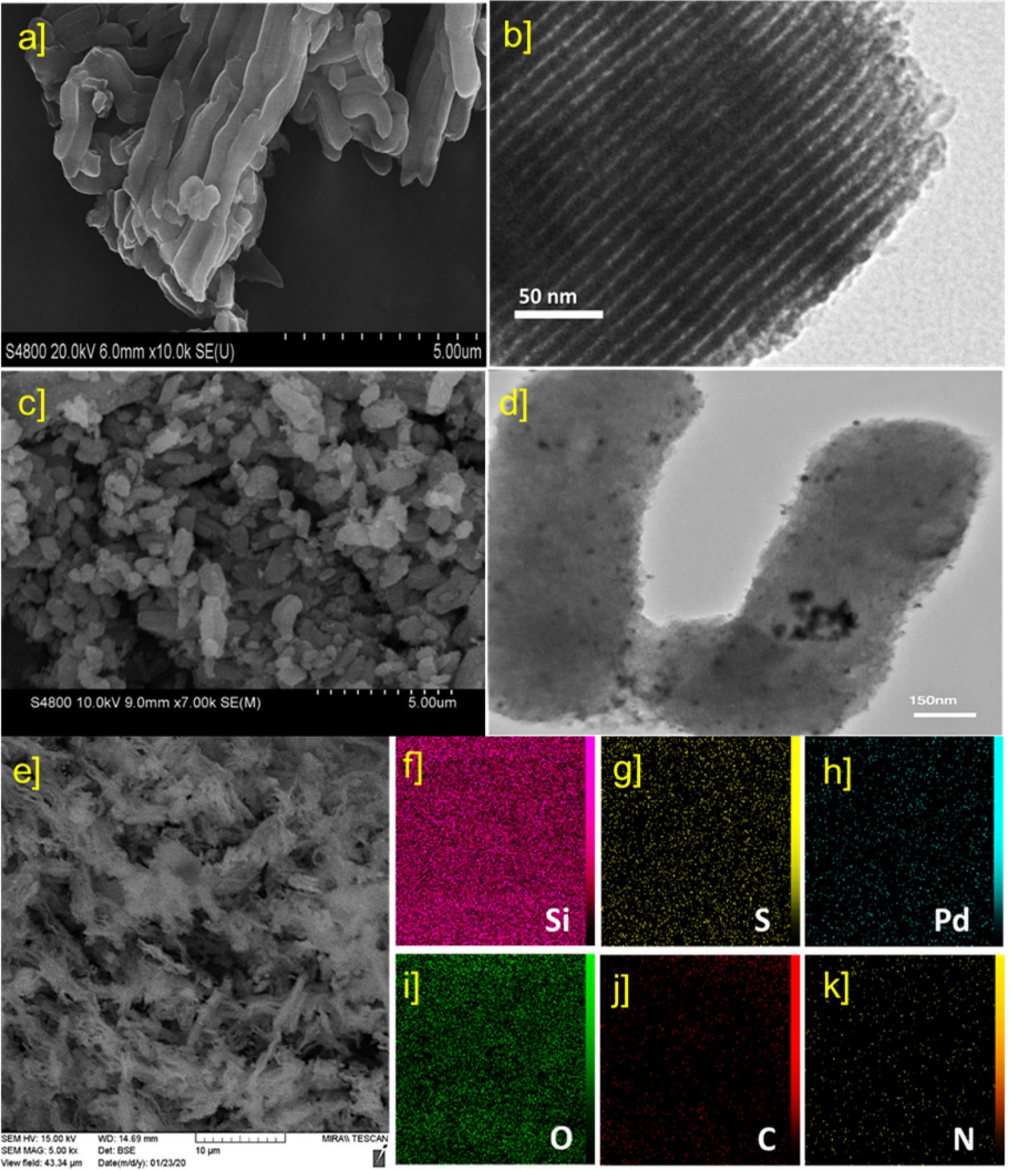
SEM images of (**a**) SBA-15/DTC and (**c**) Pd_NPs_@SBA-15/DTC. TEM images of (**b**) SBA-15/DTC and (**d**) Pd_NPs_@SBA-15/DTC. (**e**) STEM-EDS and (**f**–**k**) elemental mappings of Pd_NPs_@SBA-15/DTC.

The FE-SEM image of the fabricated SBA-15/DTC indicates a rod-like morphology Fig. 3a. After deposition of Pd NPs on SBA-15/DTC surface, the rod-like structures preserve, which demonstrated NPs were orderly anchored (Fig. 3b,c). The TEM images further reveal the spherical Pd NPs that are well-distributed on the surface of the SBA-15/DTC Fig. 3d. The surface of mesoporous silica with DTC ligand helps for the stabilization of Pd NPs and inhibition of agglomeration. The STEM-EDS Fig. 3e and elemental mapping images in Fig. 3f-k proved the existence of all constituent elements (i.e., Si, O, C, N, S, Pd) in the mesoporous silica structure. It demonstrates the uniform distribution of Pd NPs in SBA-15/DTC channels.

Ultimately, after the characterization of meso-material, the catalytic activity of Pd_NPs_@SBA-15/DTC, with its comparative component for the dehydrogenation of FA at room temperature, is stated in Fig. 5a. According to Fig. 5a, 9.83 mL of gases are released in 120 min catalyzed by Pd_NPs_@SBA-15/DTC, which corresponding to an initial TOF value of 1954 and TON 3904.

To evaluate the performance of Pd_NPs_@SBA-15/DTC (2.89 wt% Pd, determined by ICP analysis), for catalytic H_2_ production, we utilize FA/SF (1: 1) as the reaction substrate. Very recently, a variety of noble metal NPs^54–56^ has been used to catalyze the selective dehydrogenation of FA under mild conditions.

**Figure 4:**
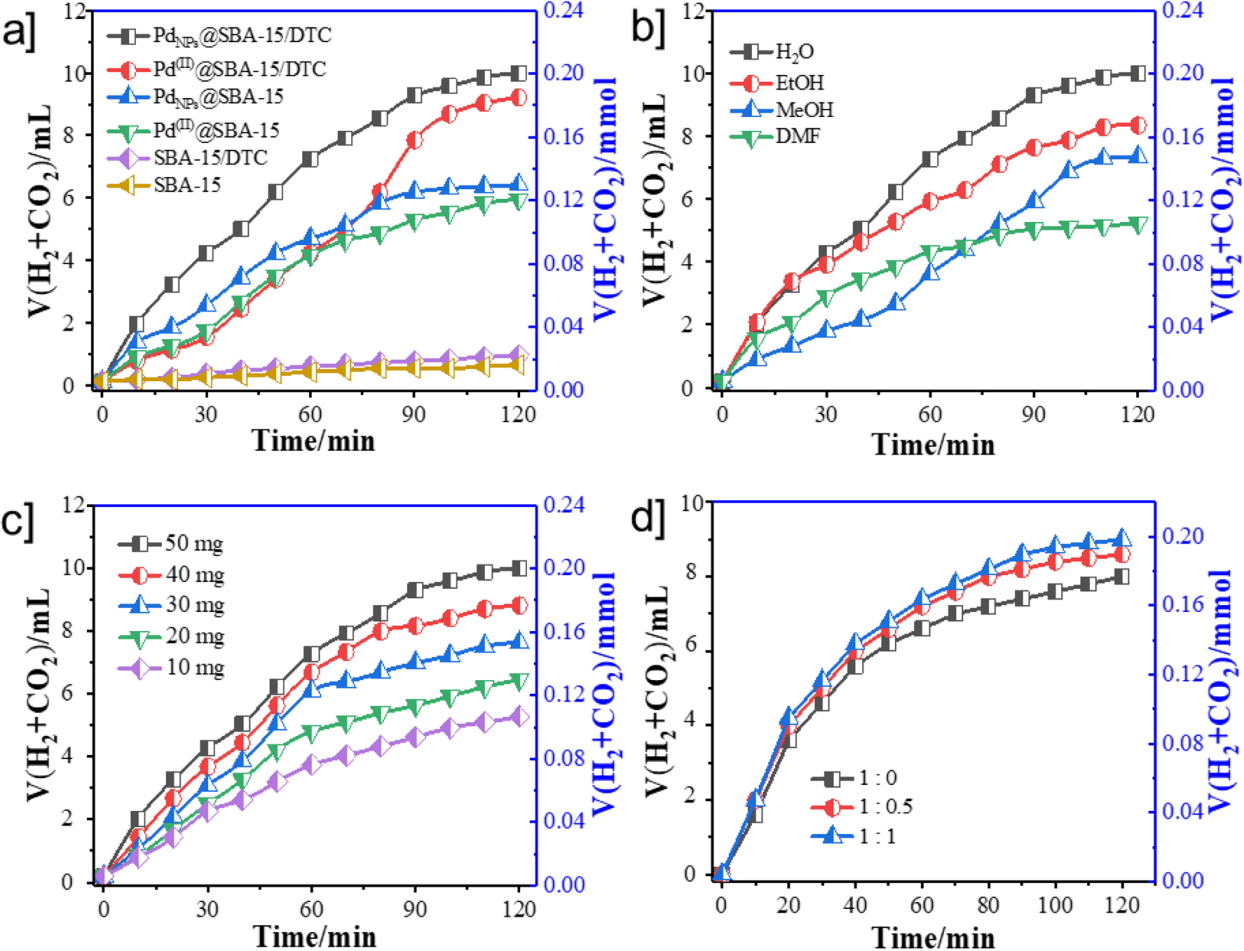
Volume of generated gas (H_2_ + CO_2_) versus time for the dehydrogenation of FA in FA-SF aqueous solution at 298 K over (**a**) different support (**b**) different solvents (**c**) amount catalyst (**d**) different mole ratio of FA/SF.

Figure 4a shows the evolution of H_2_ overtime when the Pd@SBA-15/DTC catalyst is mixed with a solution FA/SF under an ultrasonic wave. The H_2_ generation for FA complete (ca 0.205 mmol and 1954 TOF) in 120 min. Remarkably, Pd_NPs_@SBA-15/DTC had catalytic activity comparable to immobilized Pd complex (Pd^(II)^@SBA-15/DTC), Pd^(II)^@SBA-15, SBA-15/DTC, and SBA-15 without the use of any additive in presence ultrasonic wave. This result demonstrates the DTC groups can apparently elevate the electron transfer from the host to Pd NPs and thus increases activity dehydrogenation FA. Even though the H_2_ amount for Pd_NPs_@SBA-15/DTC is more than others, we continued our investigations with encapsulated Pd NPs. For instance, when we investigated the dehydrogenation progress with various solvents such as EtOH, MeOH, DMF, and H_2_O, we found that H_2_O with 9.86 mL H_2_ gas and Tenover frequency (TOF) of 1952 is more efficient than the other solvent Fig. 4b. Using Pd_NPs_@SBA-15/DTC, CO generation was insignificant (detected by GC and NaOH trap experiments), whereas H_2_ generation as well associates with *literature* data^57,58^. When the amount of the catalyst increased from 10 to 50 mg, the FA dehydrogenation was also increased (Fig. 4c). On the other hand, the catalytic activity of Pd_NPs_@SBA-15/DTC is enhanced by the addition of SF, and we continued our studies in the best mole ratio of FA: SF is 1:1 (Fig. 4d).

**Figure 5:**
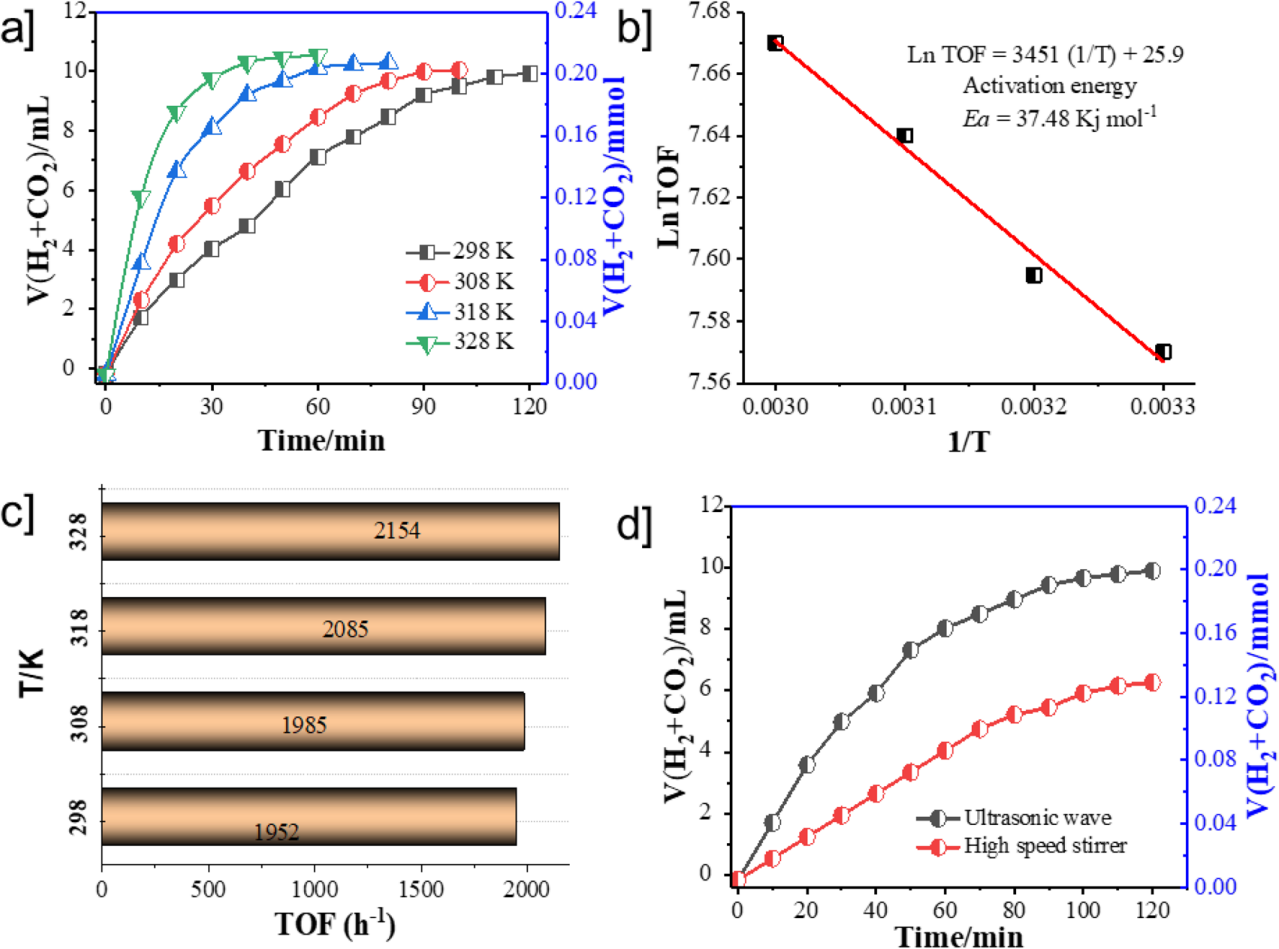
(**a**) Volume of generated gas (H_2_ + CO_2_) versus time for the dehydrogenation of FA without the addition of SF over Pd_NPs_@SBA-15/DTC at different temperature (**b**) corresponding kinetic parameters (**c**) corresponding TOF (**d**) relationship between gases production under ultrasonic irradiation or stirrer over Pd_NPs_@SBA-15/DTC.

Additionally, when the reaction was tested at various temperatures, it showed that ultrasonic condition in 335 K accelerates the decomposition of FA reaction Fig. 5a. Because of the enhanced rate of the dehydrogenation FA at room temperature and the significant importance of associated chemical processes and energy issues, we continued our studies under ambient conditions. According to this study, the activation energy (*Ea*) of the dehydrogenation FA is 37.48 ± 2.0 kJ mol^-1^ according to the Arrhenius plot, which is in agreement with previous studies Fig. 5b. The catalytic performance is related to the reaction temperature in a positive way, giving the TOF values of 1952, 1985, 2085, and 2154 h^−1^ at the corresponding reaction temperatures Fig. 5c, respectively. As represented in (Fig. 5d), the activity of all SBA-15 samples was enhanced under the wave of ultrasonic, and the Pd_NPs_@SBA-15/DTC produce a large amount of H_2_ generation in comparison with that high-speed stirrer (HSS). Therefore the suggestion is that ultrasonic irradiation is the third function that acts as a synergistic agent in the catalytic decomposition of FA.

**Figure 6:**
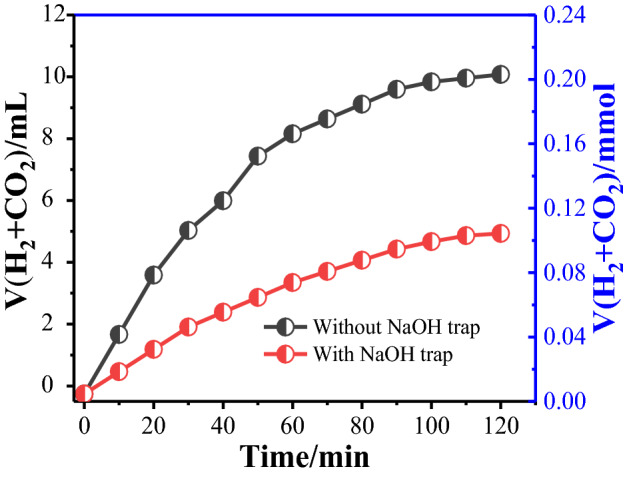
Volume of generated gases versus time for the dehydrogenation of FA in FA-SF aqueous solution at room temperature over Pd_NPs_@SBA-15/DTC with/without NaOH trap.

According to the promising results obtained above, NaOH trap experiments were performed to determine the volume ratio of H_2_ to CO_2_ in the dehydrogenation process. The produced gas was treated with/without NaOH trap (Fig. 6). The volume of produced gas was decreased to half after treating with the 10 M NaOH aqueous solution. The results demonstrate complete adsorption of CO_2_ gas in NaOH solution (I), and the produced gas is H_2_ and CO_2_ with the volume ratio of 1:1.

1 $${\text{(I)}}\;{\text{CO}}_{{2}} \left( {\text{g}} \right) + {\text{2NaOH}}\;\left( {\text{l}} \right) \to {\text{Na}}_{{2}} {\text{CO}}_{{3}} \left( {\text{s}} \right) + {\text{H}}_{{2}} {\text{O}}$$

To compare Pd-SBA-15 activity with benchmark ligands, some catalytic tests were carried out also with ligands donor electrons such as bis-thiourea (BTU), S–H and dithiocarbamate (DTC), which result presented in Table 1. In the presence of DTC ligand, 3.41% conversion was achieved in 2 h with maximum volume gases 9.86 mL, giving the highest TOF = 1952 h^−1^ in comparison with other ligands stabilize Pd NPs. The results show the high efficiency of DTC ligand compared to other ligands for stabilizing and keeping Pd inactive state in the structure of SBA-15.

**Table 1:** Results of catalytic FA dehydrogenation in the presence of various ligand.

Entry	Ligand	Metal	Max vol (mL) [t(min)]^a^	TOF (h^−1^)^b^	TON tot^c^
1	S–H	Pd^(II)^	2.26 [135]	50.8	114.3
2	S–H	Pd_NPs_	2.81 [135]	54.2	121.9
3	BTU	Pd^(II)^	5.69 [180]	40.1	120.3
4	BTU	Pd_NPs_	6.03 [180]	42.5	127.0
5	DTC	Pd^(II)^	8.76 [120]	1739	3478
6	DTC	Pd_NPs_	9.83 [120]	1952	3904

Conditions: 5 mL, 5% v/v HCOOH, 50 mg pre-catalyst, R.T, ultrasonic wave.

^a^The values are averaged from three measurements with reproducibility of $\pm$ 5%.

^b^Defined as (mmol_produced gas_/mmol_Pd_) $\times$ h^−1^.

^c^Defined as (mmol_produced gas_/mmol_Pd_).

**Figure 7:**
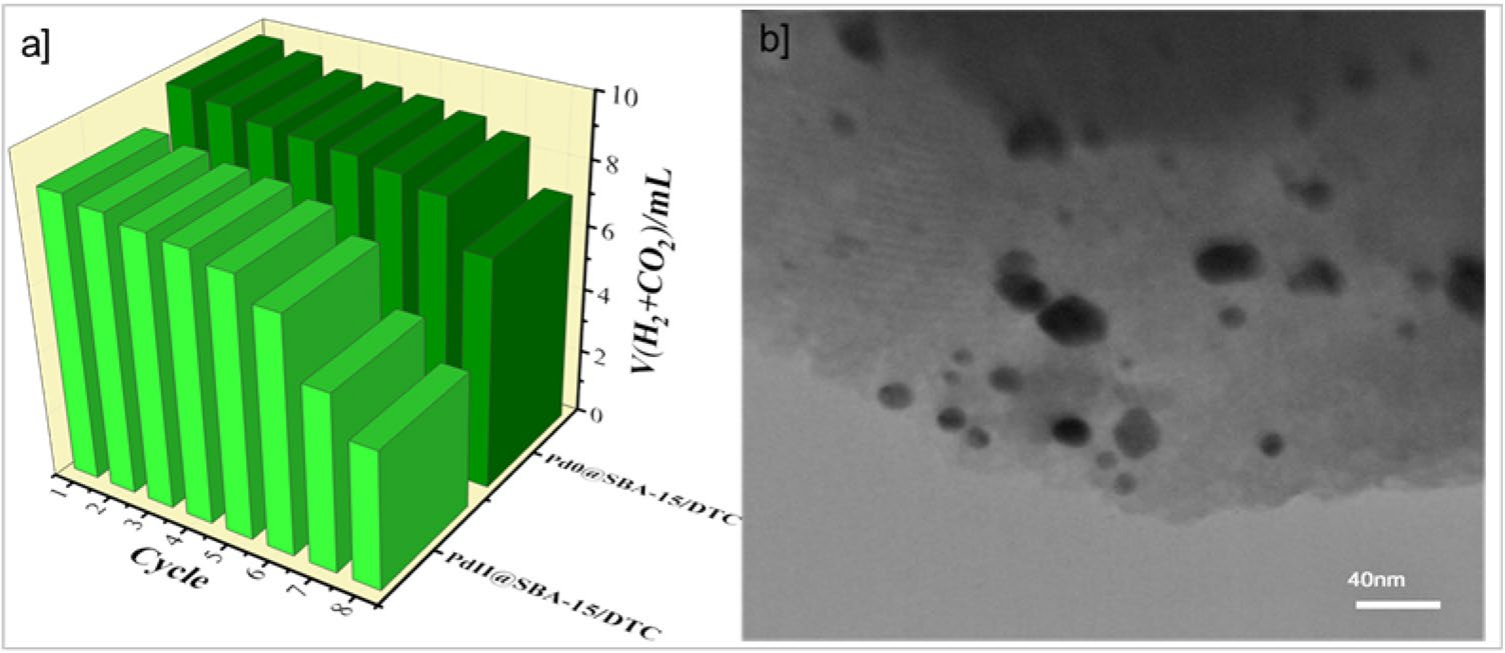
(**a**) Durability of the Pd_NPs_@SBA-15/DTC and Pd^II^@SBA-15/DTC (**b**) TEM images after the 8th recycling of Pd_NPs_@SBA-15/DTC.

Finally, runs were repeated to check the activity and stability of the Pd@SBA-15, reusability tests were also performed by adding neat FA (5% v/v) to the reaction mixtures under the ultrasonic wave. The pre-catalysts was recovered and reactivated by common filtration after each reaction. As shown in Fig. 7a, the activity was approximately kept for the first eight-time suggesting that the Pd@SBA-15 was stable during the decomposition of FA for a long time. However, after each cycle of reaction, a small amount of decrease in yield was observed, which could be attributed to the reality that a small fraction of the catalyst is lost in each recovery. Stable mesoporous silica and Pd NPs aggregation after the reuse testified by TEM images (Fig. 7b). According to these results, the proposed catalyst after 8^th^ run affirming the overall structural integrity of the material without aggregation of Pd particles.

## Conclusion

The purpose of the current study was to design DTC/SBA-15 as excellent support for anchoring and stabilizing Pd in nanoscale. The proposed catalyst shows high catalytic activity, good stability, and easy recyclability for the selective dehydrogenation of FA under ultrasonic irradiation. The results suggested that the mesoporous structure and synergistic effect of palladium metal, dithiocarbamate ligands in SBA-15 frameworks, and ultrasonic irradiation play serious roles in the catalytic performance of the H_2_ generation from FA. Moreover, by modifying this mesoporous material with the various organic ligands for stabilizing other noble metal nanoparticles can be developed to prepare versatile heterogeneous catalysts for organic transformations and environmental application.
